# Cocain

**Published:** 1885-04

**Authors:** 


### COCAIN.

Dr. Aschenbrandt, of Wurzburg, has made some experiments on the action of muriate of cocain on the human organism. He administered the drug, unknown to the subjects (who were soldiers), in doses about one-sixth of a grain in cases of exhaustion and fatigue from various causes, and found invariably that the lassitude was speedily removed, and that the men could go on for hours without feeling hunger or thirst. One of his experiments was made on himself after a sleepless night, with the prospect of a long day's march before him, when a dose of cocain (taken in coffee about 3 A. M.) enabled him to go the whole day without feeling hunger, thirst, or fatigue, and he dined late in the afternoon with his usual appetite. He considers the drug to be a direct nerve food, and not a stimulant merely; but its stimulating action is certainly far above that of alcohol, and it appears to have no injurious after effects.—*Medical Record.*

